# Skills-approximate occupations: using networks to guide jobs retraining

**DOI:** 10.1007/s41109-022-00487-7

**Published:** 2022-06-28

**Authors:** Keith Waters, Shade T. Shutters

**Affiliations:** 1 School of Complex Adaptive Systems, Arizona State University, Washington, DC USA; 2 Schar School of Policy and Government, George Mason University, Arlington, VA USA; 3 School of Complex Adaptive Systems, Arizona State University, Tempe, AZ USA; 4 Global Climate Forum, Berlin, Germany

**Keywords:** Skills, Co-occurrence, Labor retraining, Complexity theory, Occupations

## Abstract

An issue often confronting economic development agencies is how to minimize unemployment due to disruptions like technological change, trade wars, recessions, or other economic shocks. Decision makers are left to craft policies that can absorb surplus labor with as little pain to workers as possible. The questions they face include how to re-employ displaced workers and how to fill labor shortages. To address such questions, we quantify the proximity of any two occupations based on the skills inherent in each. Taking labor skills as nodes, we model US labor as a weighted network of interdependent skills, deriving link values from geographical patterns of skill co-occurrence. We use this network to locate occupations, measure their proximity to each other, and identify which missing skills may inhibit workers from easily transitioning from one occupation to another. Thus, given that an occupation is a bundle of skills, we use our skills network to help policy makers identify which other occupations are most proximate a worker’s current occupation. Finally, we apply our method to assess various worker retraining pathways for metropolitan Washington, DC, USA, whose economy was simultaneously disrupted by both the COVID-19 pandemic and the arrival of a second headquarters for Amazon.

**Supplementary Information:**

The online version contains supplementary material available at 10.1007/s41109-022-00487-7.

## Introduction

The field of economic geography has increasingly embraced a complexity science perspective to understand economic phenomena. Such studies apply techniques from complex systems science to empirical data on the geography of economic activities to characterize economies and understand development pathways. Two major themes emerging in this literature are those of relatedness metrics and complexity metrics (Hidalgo 2021). While both of these themes use methods from network analysis as a primary means of analyzing economic components of interest, relatedness approaches typically focus on one class of economic entities (e.g., industries, occupations, products, skill, etc.), creating a network, or ‘space’, to be analyzed. In such networks, nodes represent the economic entities of study (e.g., occupations) while link values are determined by some measure of proximity between pairs of nodes. The resulting network (e.g., “occupation space”) is then characterized and analyzed to better understand economic phenomena of interest.

Among the earliest and most influential of these studies was the creation and characterization of a global “product space” (Hidalgo C. A. et al. 2007). In this network nodes represent products or goods and proximity of two products *i* and *j* is defined as the conditional probability that a country exports *i* given that it exports *j*. The authors found the network to be heterogenous, with more complex goods located near the network’s core and less complex goods located primarily near its periphery. The authors go on to suggest that countries exporting those products found primarily at the network’s periphery would have difficulty transitioning into the production and export of the more complex goods found in the network’s core.

Networks – or spaces – of other economic entities soon followed. Industry space has been mapped using link values based on the co-occurrence of products in manufacturing plants (Neffke et al. 2011) and inter-industry labor flows (Neffke and Henning 2013). Knowledge space has been mapped with link values derived from the co-occurrence patterns of patent technology classes across patent applications (Kogler et al. 2013) while technology space has been mapped based on the conditional probability that a region specializes in a given patent technology class (Boschma et al. 2015). Similarly, occupation space has been mapped with link values derived from co-occurrence patterns of occupations across metropolitan areas (Muneepeerakul et al. 2013; Farinha et al. 2019). More recently, skills space has been mapped with link values derived from the co-occurrence patterns of skills both across occupations (Alabdulkareem et al. 2018) and across metropolitan areas (Shutters and Waters 2020).

As this body of scholarly literature has grown over the last 15 years, regional economic development agencies have simultaneously faced an increasing urgency to grow and protect jobs. In a survey of US elected officials, nearly half indicated that growing jobs was their top priority (Bartik 2003). Similarly, in a 2016 report from the US President’s Office, “finding and acquiring a good job, a quality education, and appropriate training” was listed first among challenges facing Americans (PCAST 2016, p. 8).

Thus, there exists a clear need and opportunity to translate the growing body of network-framed studies in economic geography into applications and tools that can inform regional economic policy.

Here, we explore this opportunity by translating recent findings into a policy-relevant decision support metric and applying it to a regional economy case study. We use recently developed metrics to quantify the skills proximity between occupations, thereby informing policy makers of which occupations could most easily absorb a surplus of workers in occupation *X* or which occupations could most easily be a source of workers for needed occupation *Y*. The first of these metrics, called transition potential, was originally created to measure the proximity between a single occupation and the full set of occupations present in a given economy (Muneepeerakul et al. 2013). Here, we use this formulation to instead measure the proximity between a single skill and the full set of skills present in a given occupation. The second metric, originally developed to measure the proximity of a starting economy and a target economy by averaging occupational transition potentials (Shutters et al. 2016), is modified here to average the transition potentials between the skills present in starting occupation and those in a target occupation. We take this aggregated transition potential to be a measure of the proximity of two occupations.

Our approach can be used to inform policy in at least three ways.

- First, for policy makers seeking to re-employ displaced workers, such as those laid-off when a coal-mine closes, proximity can be used to identify other occupations to which unemployed workers could most easily transition, given the skills profile of their previous occupation.
- Second, for policy makers facing a critical shortage of workers in a particular occupation, proximity can be used to identify other occupations that are most similar and thus offer the most efficient source of workers that could be retrained to undertake jobs in the high-demand occupation.
- Third, for policy makers addressing either of the transitions described above, our network-metrics can identify which skills are likely to be the largest obstacles to successful retraining, enabling training program to prioritize curricula options.

In any case, proximity alone should not be used to prioritize new job targets but should be used in conjunction with numerous other factors such as an occupation’s wages, its anticipated future demand, and its fit with local long-term vision. Thus, our network-based measures are meant to augment the suite of information available to decision makers not to replace them.

### Application case study: The regional economy of metropolitan Washington, DC

To illustrate how our approach might be used by policy makers, we apply our methodology to the economic situation in the US metropolitan statistical area (MSA) of Washington, DC. This regional economy has been recently impacted simultaneous by two major disruptions affecting its labor force.

First, the COVID-19 pandemic led to a rapid and dramatic decline in the region’s leisure and hospitality employment, which is supported by Washington’s numerous well-known tourist attractions and museums. From April 2019 to April 2020, employment in the MSA’s leisure and hospitality sector decreased from 336,000 in April 2019 to 158,000 in April 2020, a decline of 52.9%. The sector recovered somewhat by December 2020 with employment down 32.5% compared to December 2019 (U.S. Bureau of Labor Statistics 2022).

Second, in November 2018, Amazon announced it would build its second headquarters in metropolitan Washington, DC, and expected to create more than 25,000 technical and administration jobs. Technology workers were already scarce before the announcement, but once Amazon began hiring for computer-related occupations, the availability of qualified workers became scarcer.

Thus, the Washington, DC regional economy faces the dual issues of high unemployment among leisure and hospitality workers and a severe shortage of qualified workers for computer-related occupations, and offers an ideal case for which to demonstrate the applicability of our methodology.

## Data and methods

We use two publicly available datasets to conduct our analysis. The first is the O*NET dataset published by the Occupational Information Network. O*NET data decomposes US occupations *o* into several hundred characteristics which are referred to as “elements” *i*. For each of 161 elements a value of level *l*_*i,o*_ ∈ (0, 7) is assigned for each occupation, indicating the degree to which the element is required or needed to perform the activities of that occupation (National Center for O*NET Development 2020). We use O*NET version 24.2 to obtain level values for each element-occupation pair. O*Net groups elements into a number of categories such as skills, work activities, abilities, and knowledge. However, it has become conventional in studies using O*NET elements to refer to all elements, regardless of category, simply as skills (e.g., Florida et al. 2012; Kok and Weel 2014; Alabdulkareem et al. 2018; Deming and Noray 2018; Vona et al. 2018; Farinha et al. 2019). We adopt this convention and hereafter refer to all O*NET elements simply as skills.

The second dataset we use is the Occupational Employment and Wage Statistics (OEWS) dataset published by the US Bureau of Labor Statistics (BLS). The OEWS dataset is published annually each May and provides estimates of employment and wages by occupation for various US geographies, including nation, state, and metropolitan statistical area. In this study we use the May 2018 dataset (U.S. Bureau of Labor Statistics 2018).

Note that O*NET uses an 8-digit occupation code while the OEWS reports employment using the more aggregated 6-digit Standard Occupation Coding (SOC) code. Thus, for each a few OEWS occupation there exist multiple corresponding occupations in O*NET data. In those cases, we use simple average to collapse the skill values of the multiple O*NET occupations into a single value for the corresponding OEWS occupation code following previous literature (Shutters and Waters 2020). See Additional files 1 and 2 for more detail. 

We use only employment data for US metropolitan statistical areas (MSAs). MSAs are defined as cohesive regional economic units comprising one or more counties, based primarily on commuting patterns. Note that, while the BLS states that OEWS data is aggregated by MSA, in the six states comprising New England the data are actually aggregated by an alternative geographic unit known as New England City and Town Areas (NECTAs). Hereafter, we adopt the convention of the OEWS data and refer to both MSAs and NECTAs collectively as MSAs.

### Constructing the skills network

Given the set of skills *i* defined by O*NET, we construct a skills network $\mathcal{G}=\left(\mathcal{N},\mathcal{L}\right),$ with nodes $\mathcal{N}=\left\{{i}_{1}, {i}_{2},\dots {, i}_{161}\right\}$. The network is complete and undirected, with the matrix of link values **X** derived from co-occurrence patterns of skills across MSAs as follows. Following Shutters and Waters (2020), we first measure the aggregate level *s* of each skill *i* in each MSA *m* as

1 $$s_{i,m} = \mathop \sum \limits_{o} l_{i,o} w_{o,m}$$

where ${l}_{i,o}$ is the level of skill *i* in occupation *o* taken from the O*NET dataset, and *w*_*o,m*_ is the number of workers *w* employed in occupation *o* in MSA *m* taken from the OEWS dataset. To standardize across MSAs of different sizes we take the location quotient or *LQ* (Isard 1960; Treyz 1993; Hidalgo 2021) of each skill in each MSA as

2 $$LQ_{i,m} = \frac{{\left( { s_{i,m} /\mathop \sum \nolimits_{i} s_{i,m} } \right)}}{{\left( {\mathop \sum \nolimits_{m} s_{i,m} /\mathop \sum \nolimits_{m} \mathop \sum \nolimits_{i} s_{i,m} } \right)}} .$$

The *LQ* value, which is also referred to in related literature as Relative Comparative Advantage or RCA (e.g. Alabdulkareem et al. 2018), is then used to designate each skill *i* as either present or absent in each MSA *m* such that if *LQ*_*i,m*_ ≥ 1, *i* is considered present in *m*, and if *LQ*_*i,m*_ < 1, *i* is considered absent in *m*. The co-occurrence patterns of present/absent skills across MSAs are then used to quantify an interdependence *x* between each pair of skills *i* and *j* as

3 $$x_{i,j} = \frac{{P\left[ {LQ_{i,m} > 1,LQ_{j,m} > 1} \right]}}{{P\left[ {LQ_{{i,m^{\prime}}} > 1} \right]P\left[ {LQ_{{j,m^{\prime\prime}}} > 1} \right]}} - 1,$$

where *m*, *m’* and *m”* are randomly selected MSAs. Thus, interdependence *x*_*i,j*_ is the conditional probability that *i* and *j* are present in the same MSA divided by the product of their marginal probabilities of being present in a random MSA. The result is a symmetric skill $\times$ skill matrix **X** which we take as the values of the links $\mathcal{L}$ in the skills network $\mathcal{G}$. Skill pairs that co-locate in MSAs more often than expected by chance have *x*_*i,j*_ > 0 while pairs that co-locate less often than expected by chance have *x*_*i,j*_ < 0. Self-links are ignored.

### Locating individual occupations in the skills network

Having constructed the full US skills network $\mathcal{G}$, we next identify the “location” of each occupation *o* within that network as the subnetwork of skills present in *o*: ${\mathcal{G}}_{o}=({\mathcal{N}}_{o}, {\mathcal{L}}_{o})$ where ${\mathcal{N}}_{o}$ $\subseteq \mathcal{N}$ includes only the skills *i* that are present in occupation *o*, and ${\mathcal{L}}_{o}\subseteq \mathcal{L}$ includes only the links between the members of ${\mathcal{N}}_{o}.$ Nearly every skill has some positive value for every occupation and thus, to make a meaningful determination of which skills are relevant to each occupation, we again use a location quotient to designate each skill *i* as either present or absent within a particular occupation *o* as

4 $$LQ_{i,o} = \frac{{\left( { l_{i,o} w_{o} /\mathop \sum \nolimits_{i} l_{i,o} w_{o} } \right)}}{{\left( {\mathop \sum \nolimits_{o} l_{i,o} w_{o} /\mathop \sum \nolimits_{o} \mathop \sum \nolimits_{i} l_{i,o} w_{o} } \right)}} .$$

where ${l}_{i,o}$ is the level of skill *i* in occupation *o* and ${w}_{o}$ is the total employment in occupation *o* across all MSAs. We take *LQ*_*i,o*_ ≥ 1 to indicate that skill *i* is present in occupation *o* and *LQ*_*i,o*_ < 1 to indicate that *i* is absent in *o.* Thus, ${\mathcal{N}}_{o}$ is the set of all skills in occupation *o* for which *LQ*_*i,o*_ ≥ 1. Note that Eq. (4) is the *LQ* of skills across occupations, while Eq. (2) is the *LQ* of skills across MSAs.

### Measuring proximity between occupations

Having defined the full skills network $\mathcal{G}$ and the location of each occupation *o* as a sub-network ${\mathcal{G}}_{o}$, we next adapt a measure of proximity between ${\mathcal{G}}_{o}$ and a single skill *i* known as the transition potential ${V}_{i}({\mathcal{G}}_{o})$(Muneepeerakul et al. 2013)*.* If skill *i* is not present in occupation *o* $\left(i\notin {\mathcal{N}}_{o}\right)$, the transition potential of *i* is calculated as

5 $$V_{{i \notin {\mathcal{N}}_{o} }} \left( {{\mathcal{G}}_{o} } \right) = 1 - \mathop \prod \limits_{{j \in {\mathcal{N}}_{o} }} \left( {1 - c\left( {1 + x_{i,j} } \right)P\left[ {LQ_{i} > 1} \right]} \right)$$

where *i* and *j* are different skills, *x*_*i,j*_ is the link value between *i* and *j*, *LQ*_*i*_ is the probability that a skill is present in a random occupation, and *c* is an arbitrary scaling parameter which we set to *c* = 0.002 for consistency with Muneepeerakul et al. (2013). For skills *i* that are present in occupation *o*, by definition transition potential ${V}_{i\in {\mathcal{N}}_{o}}\left({\mathcal{G}}_{o}\right)= 1.$

Finally, using an adapted version of the creative jobs index developed in Shutters et al. (2016), we calculate the average transition potential between each skill *i* present in target occupation *T* and the subnetwork of starting occupation ${\mathcal{G}}_{S}$. We define this average as ${P}_{S\to T}$, the proximity of starting occupation *S* to target occupation *T*:

6 $$P_{S \to T} = \frac{1}{{n_{T} }}\mathop \sum \limits_{{i \in {\mathcal{N}}_{T} }} V_{i} \left( {{\mathcal{G}}_{S} } \right)^{{1 - \delta_{i} }}$$

where *i* is a skill present in target occupation *T*, ${n}_{T}$ is the total number of skills present in *T*, and *δ* is a binary indicator such that *δ* = 1 if ${i\in \mathcal{N}}_{S}$ and *δ* = 0 if $i{\notin \mathcal{N}}_{S}$. Thus, *δ* sets the transition potential to *V*_*i*_ = 1 if skill ${i\in \mathcal{N}}_{T}$ is already present in the starting occupation *S*.

It is important to note that proximity *P* is directional, meaning that ${P}_{S\to T}$ does not necessarily equal ${P}_{T\to S}$. To illustrate, consider two occupations: *a* comprised of 100 skills, and *b* comprised of 10 skills, all of which are also present in *a*. A worker transitioning from occupation *b* to *a* must acquire 90 new skills while a worker transitioning from *a* to *b* already possesses all required skills. Thus, ${P}_{b\to a}$ is likely much lower than ${P}_{a\to b}$_._

## Results

### Skills interdependence

Using Eq. (3) we derive an interdependence value *x* between each pair of skills *i* and *j*. We take the full set of interdependence values to be the matrix of the link values **X** in our US skills network. Note that interdependence is symmetric so that *x*_*i,j*_ = *x*_*j,i*_. Self-links are ignored and *x*_*i,i*_ = 0 for all *i*. Skill pairs having the five highest interdependence values are provided in Table 1. Thus, the skills “Complex Problem Solving” and “Thinking Creatively” have the strongest co-occurrence pattern of any skill pair, meaning that it is quite rare for an MSA to include one of these skills without the other.

**Table 1:** Five highest skill-pair interdependence values (${x}_{i,j}$)

Rank	Skill *i*	Skill *j*	$\varvec{x}_{{\varvec{i,j}}}$
1	Complex problem solving	Thinking creatively	4.887
2	Complex problem solving	Judgment and decision making	4.579
3	Making decisions and solving problems	Thinking creatively	4.562
4	Thinking creatively	Provide consultation and advice to others	4.533
5	Thinking creatively	Developing objectives and strategies	4.508

### Skills-based occupation proximity

**Fig. 1:**
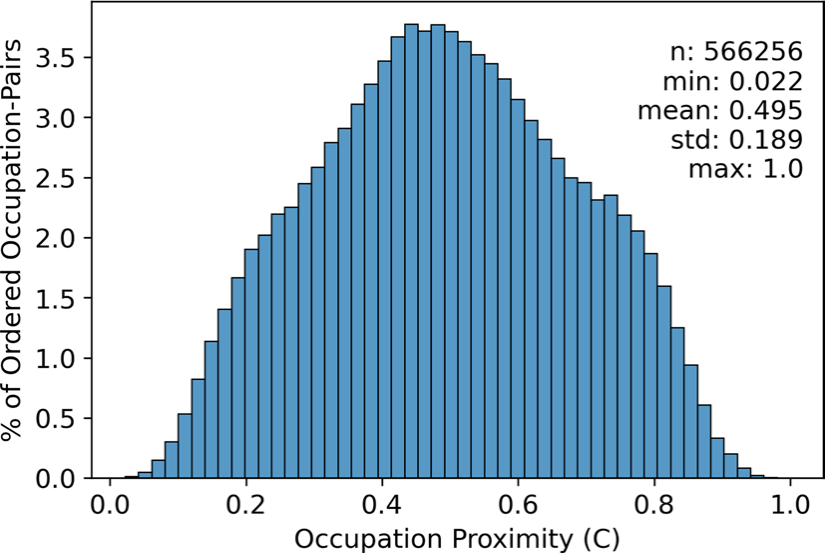
Distribution of occupational proximities ${P}_{S\to T}$. The distribution is approximately symmetric with mean = 0.45 and standard deviation = 0.189. Note that each occupation pair will have two proximities depending on which is the starting occupation S and which is the target T

We next apply Eqs. (4)–(6) to derive proximity ${P}_{S\to T}$ between each of 566,256 ordered pairs of US occupations, one of which in each case is the starting occupation *S* and the other is the target occupation *T*. The distribution of these proximities (Fig. 1) is roughly symmetric with mean = 0.495 and standard deviation = 0.189. Occupational pairs having the five highest and lowest proximities are presented in Table 2. Note that for three occupation pairs ${P}_{S\to T}$= 1, meaning that the starting occupation in each case already possess all the skills required by the target occupation. Two of these pairs are reciprocals of each other, “Substitute Teachers” and “Teachers and Instructors, All Other, Except Substitute Teachers”, meaning these two occupations are composed of the same set of skills.

**Table 2:** Highest five and lowest five occupation proximity values ${P}_{S\to T}$ between occupation pairs

Proximity rank	$\varvec{P}_{{\varvec{S} \to \varvec{T}}}$	Starting occupation *S*	Target occupation *T*
1	1.000	Teachers and instructors, all other, except substitute teachers (25–3097)	Substitute teachers (25–3098)
2	1.000	Substitute teachers (25–3098)	Teachers and instructors, all other, except substitute teachers (25–3097)
3	1.000	Woodworking machine setters, operators, and tenders, except sawing (51–7042)	Molding, coremaking, and casting machine setters, operators, and tenders, metal and plastic (51–4072)
4	0.985	Installation, maintenance, and repair workers, all other (49–9099)	Mobile heavy equipment mechanics, except engines (49–3042)
5	0.984	Installation, maintenance, and repair workers, all other (49–9099)	Wind turbine service technicians (49–9081)
…	…	…	…
566,252	0.024	Mechanical door repairers (49–9011)	Political science teachers, postsecondary (25–1065)
566,253	0.024	Bus and truck mechanics and diesel engine specialists (49–3031)	English language and literature teachers, postsecondary (25–1123)
566,254	0.023	Mobile heavy equipment mechanics, except engines (49–3042)	Law teachers, postsecondary (25–1112)
566,255	0.023	Rail car repairers (49–3043)	Law teachers, postsecondary (25–1112)
566,256	0.022	Bus and truck mechanics and diesel engine specialists (49–3031)	Law teachers, postsecondary (25–1112)

On the other hand, when “Woodworking Machine Setters, Operators, and Tenders, Except Sawing” is the starting occupation *S*, it already possesses all 59 skills present in the target “Molding, Coremaking, and Casting Machine Setters, Operators, and Tenders, Metal and Plastic” *T*, meaning that ${P}_{S\to T}=1$. However, the reverse is not true as “Woodworking Machine Setters, Operators, and Tenders, Except Sawing” has 12 additional skills and ${P}_{T\to S} = 0.838$. The lowest proximity value occurs when the starting occupation is “Labor Relations Specialists” and the target is “Molding, Coremaking, and Casting Machine Setters “. Thus, workers seeking to make this transition would likely face one of the most difficult retraining pathways of any transition.

We reiterate three possible uses for our methodology:

- To identify other occupations to which unemployed workers could most easily transition,
- To identify other occupations that may most efficiently supply labor for an occupation having a shortage of workers, and
- To identify which skills that are likely to be significant obstacles to successful retraining, enabling training program to prioritize curricula options.

With these uses in mind we turn to a case study to illustrate how our methodology might be applied in an actual policy setting.

## Case study: Washington, DC metropolitan statistical area (MSA)

Due to the nearly simultaneous disruptions of the COVID-19 pandemic fallout and being chosen as the site of Amazon’s second headquarters, the Washington, DC MSA faces the dual issues of high unemployment among leisure and hospitality workers and a severe shortage of qualified workers for computer-related jobs. One possible strategy by policy makers is to retrain some of the surplus of unemployed hospitality workers so that they may fill some of the many vacant computer-related jobs.

For our demonstration case study, we use standard occupations “Waiters and Waitresses” (SOC 35–3031) as the starting occupation and both “Computer User Support Specialists” (SOC 15–1151) and “Computer Network Support Specialists” (SOC 15–1152) as target occupations. Using the network-based proximities of “Waiters and Waitresses” to both targets, we inform potential policy decisions regarding possible retraining pathways. Furthermore, we use proximities of two computer occupations *from* all others to identify workers besides “Waiters and Waitresses” that may be a quality source of workers for high-demand computer-related jobs.

### Assessing targets, given a starting occupation

Given that workers are displaced from starting occupation *S* = “Waiters and Waitresses”, we compare the proximity from *S* to two possible target occupations *T*. When *T* is “Computer User Support Specialists” ${P}_{S\to T}$= 0.388, while when *T* is “Computer Network Support Specialists” ${P}_{S\to T}$= 0.273. Thus, our proximity suggests that, between the two target occupations, “Waiters and Waitresses” would require less retraining to transition to “Computer User Support Specialists” than to “Computer Network Support Specialists”.

Note, however, that the occupational proximities of both targets in this example are less than the mean ${P}_{S\to T}$ of 0.495. This suggests that transitioning from “Waiters and Waitresses” to either computer-related occupation may be more difficult than transitioning to many other occupations. Thus, we can use our approach to further inform policy makers of target occupations having high proximity as alternatives to computer-related jobs. Table 3 presents five target occupations having the highest proximity to “Waiters and Waitresses” along with the occupations’ 2018 mean annual wages and *LQ*s for the Washington, DC MSA.

**Table 3:** Occupations most proximate to starting occupation “Waiters and Waitresses”, with mean annual wages and *LQ* values for the Washington, DC MSA

Target Occupation (SOC)	$$\varvec{P}_{{\varvec{S} \to \varvec{T}}}$$	Mean annual wages*	LQ*
Dining Room/Cafeteria Attendants and Bartender Helpers (35–9011)	0.696	$28,410	1.25
Orderlies (31–1015)	0.692	$30,080	0.72
Ushers, Lobby Attendants, and Ticket Takers (39–3031)	0.682	$25,000	0.85
Food Servers, Nonrestaurant (35–3041)	0.673	$27,880	0.88
Flight Attendants (53–2031)	0.660	~	~

^*^Taken from the 2018 OEWS by MSA (U.S. Bureau of Labor Statistics 2018) ~ Data not disclosed for Washington, DC MSA

While Table 3 is insightful, policy makers typically must simultaneously address multiple objectives when evaluating policy options. For example, policy makers seeking to lower unemployment, may also seek to increase average wages, protect against automation, and attract jobs with high growth potential. Thus, we present alternative results for the case of “Waiters and Waitresses” in Washington, DC, but highlight occupations that have a combination of both high proximity and high wages. Table 4 lists the five occupations with highest proximity to “Waiters and Waitresses” that also earn more than $75,000 in annual wages in the Washington, DC MSA. Thus, “Waiters and Waitresses” would likely require more retraining to transition to “Nurse Midwives” than they would to “Orderlies”, but they would earn significantly more in wages. Our approach does not optimize across multiple objectives but instead gives policy makers critical information so they can navigate these tradeoffs more easily.

**Table 4:** Occupations most proximate to starting occupation “Waiters and Waitresses” having annual wages greater than $75,000 for the Washington, DC MSA

Target occupation (SOC)	$$\varvec{P}_{{\varvec{S} \to \varvec{T}}}$$	Mean annual wages*
Nurse Midwives (29–1161)	0.569	$101,400
Nurse Practitioners (29–1171)	0.545	$112,330
Special Education Teachers, All Other (25–2059)	0.531	$75,250
Healthcare Practitioners and Technical Workers, All Other (29–9099)	0.525	$97,970
Locomotive Engineers (53–4011)	0.515	$78,140

^*^Taken from the 2018 OEWS by MSA (U.S. Bureau of Labor Statistics 2018)

### Assessing starting occupation, given a target

In addition to the need to re-employ displace workers, policy makers may face scenarios in which critical target occupations go unfilled in the local economy. These vacancies may, for example, be hindering growth in important industries or preventing the establishment of new industries. One possible policy option to address this situation is to incentivize workers in existing occupations that could be efficiently retrained for the target occupation. Here we use our methodology to identify starting occupations with high proximity to a target occupation. We again use “Computer User Support Specialists” which are in critical demand in the Washington DC, MSA exacerbated by the introduction of a new Amazon headquarters. Table 5 lists the five starting occupations *S* with highest proximity to “Computer User Support Specialists” *T*. Thus, in our example, policy makers may wish to inform existing workers in jobs such as “Biological Technicians” and “Information Security Analysts” about career opportunities in computer-related occupations.

**Table 5:** Starting occupations with five highest proximity values to target occupation “Computer User Support Specialists”

Starting occupation (SOC)	$\varvec{P}_{{\varvec{S} \to \varvec{T}}}$
Biological technicians (19–4021)	0.791
Information security analysts (15–1122)	0.791
Film and video editors (27–4032)	0.768
Microbiologists (19–1022)	0.768
Biochemists and biophysicists (19–1021)	0.755

### Identifying skills likely to be problematic for a worker transition

**Fig. 2:**
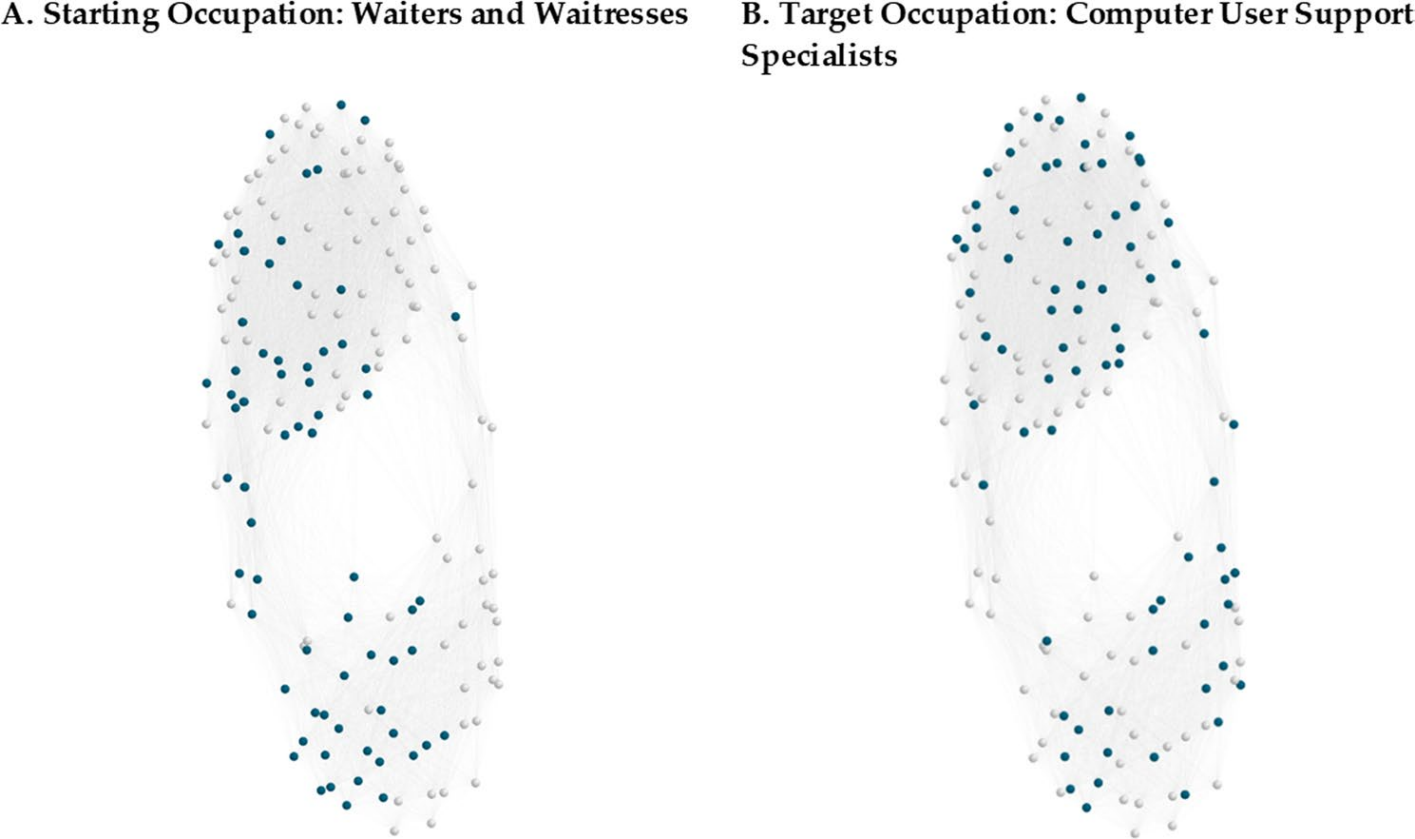
Occupation locations of (**A**) ${\mathcal{N}}_{S}$ and (**B**) ${\mathcal{N}}_{T}$ within the full skills network $\mathcal{G}.$ Here the starting occupation *S* is “Waiters and Waitresses” and the target occupation *T* is “Computer User Support Specialist”. Skills present in ${\mathcal{N}}_{S}$ and ${\mathcal{N}}_{T}$ are highlighted while those absent are greyed out. Though negative link values are used to calculate occupational proximity ${P}_{S\to T}$, they are excluded from these visualizations, which were rendered in Pajek using the Kamada-Kawaii algorithm

To identify skills that may present the largest obstacles to a worker transition, we compare the transition potentials (Eq. 5) of each skill that is missing in a starting occupation *S* but is required in the target occupation *T*. Again, we use “Waiters and Waitresses” as the starting occupation *S* and “Computer User Support Specialists” as the target *T*. The skills present in each of these occupations are highlighted in Fig. 2 as sets of nodes ${\mathcal{N}}_{S}$ and ${\mathcal{N}}_{T}$ within the full US skills network $\mathcal{G}$. Links with negative values are excluded for clarity and visualizations were prepared with Pajek using the Kamada-Kawaii algorithm (Mrvar and Batagelj).

Among skills required to transition from “Waiters and Waitresses” to “Computer User Support Specialists”, those with the highest and lowest transition potentials ${V}_{i\in {\mathcal{N}}_{T}}\left({\mathcal{G}}_{s}\right)$ are shown in Table 6. This ignores skills required in the target occupation that are already present in the starting occupation, meaning ${V}_{i}\left({\mathcal{G}}_{s}\right)=1$. Skills more likely to hinder a transition are “Thinking Creatively” and “Complex Problem Solving”, which have low transition potentials. These skills would likely require more emphasis in a worker retraining program. In contrast, “Waiters and Waitresses” would likely require less training (if any) in “Public Safety and Security” and “Wrist-Finger Speed”. These skills represent capabilities of “Waiters and Waitresses” that could help facilitate a transition to “Computer User Support Specialists”.

**Table 6:** Skill transition potentials from waiters and waitresses *S* to computer user support specialists *T*

Rank	Skill $i\in {\mathcal{N}}_{T}$	${V}_{i}\left({\mathcal{G}}_{s}\right)$
1	Public safety and security	0.0991
2	Wrist-finger speed	0.0939
3	Repairing and maintaining mechanical equipment	0.0813
4	Operation and control	0.0804
5	Operation monitoring	0.0704
…	…	…
44	Interpreting the meaning of information for others	0.0271
45	Programming	0.0261
46	Telecommunications	0.0260
47	Complex problem solving	0.0258
48	Thinking creatively	0.0247

### Limitations of our approach and future directions

At its most fundamental level, our approach depends on measuring similarity of networks, particularly subnetworks within a parent network. However, there is no universally accepted best method of calculating such measures. In our case the links of those networks have non-binary values including negative values, which complicates measures of similarity. In addition, the nodes of our networks also have non-binary values, though in this study we collapse those values into a binary present or absent designation. Thus, there is much research to be done on the best ways to measure similarity of networks, including those that have link and node values. It is highly likely that improvements to such measures will also improve our ability to use economic networks to inform policy makers.

## Conclusion

Here we present a network-based methodology for identifying the proximity of two occupations given the skills present in each. We propose that this methodology may help inform regional economic developers concerned with minimizing labor disruptions due, for instance, to a surplus of unemployed workers or a shortage of workers for needed occupations. We demonstrate the potential utility of this approach through an application to a case study of the US metropolitan area of Washington, DC, which has recently experienced significant economic disruption. We demonstrate, in this case, how policy makers may prioritize targets for re-employment of displaced workers and how they might identify specific skills that require added emphasis in worker-retraining programs. While we certainly would not suggest that our method replace existing approaches to economic development, we believe it does additional and new information, and would thus fit well into the toolbox of a policy maker seeking to efficiently guide a regional economy through a disruption or transformation.

## Supplementary Information

**Additional file 1:** This file describes how the O*NET data are mapped to BLS data.

**Additional file 2:** This file provides the full mapping from O*NET to BLS data.

## Data Availability

Data used in this study are publicly available from O*NET at https://www.onetcenter.org/db_releases.html and the US Bureau of Labor Statistics at https://www.bls.gov/oes/tables.htm.

## Abbreviations

BLS: Bureau of labor statistics
MSA: Metropolitan statistical area
NAICS: North American industrial classification system
O*NET: Occupational information network
OEWS: Occupational employment and wage statistics
SOC: Standard occupation classifications
NECTA: New England city and town area
LQ: Location quotient
