# Supplementary material for: Skills-approximate occupations: using networks to guide jobs retraining
Source: Appl Netw Sci. 2022 Jun 28;7(1):43. doi: 10.1007/s41109-022-00487-7 (PMC9244569; doi:10.1007/s41109-022-00487-7)
Supplement: Supplementary file 1 — Additional file 1: This file describes how the O*NET data are mapped to BLS data. [file 41109_2022_487_MOESM1_ESM.docx]

**Supplementary Materials for**

Skills-Approximate Occupations: Using Networks to Guide Jobs Retraining

Keith Waters^1,*^ and Shade T. Shutters ^2^

^1^ Arizona State University; [shade.shutters@asu.edu](mailto:shade.shutters@asu.edu)

^2^ Arizona State University; [keith.waters@asu.edu](mailto:keith.waters@asu.edu)

***** Correspondence: [keith.waters@asu.edu](mailto:keith.waters@asu.edu)

**S1. Mapping O*NET occupation codes to BLS occupation codes**

In our analysis we paired O*NET version 24.2 (1) with the Bureau of Labor Statistics (BLS) 2018 occupational employment statistics (2). These two entities use slightly different occupation codes. In particular, the BLS uses the federal standard 6-digit code, while O*NET adds an extra 2-digits so that occupations may be further divided. Here we describe in detail our procedure for linking the two data sets.

With two exceptions, every O*NET occupation code can be mapped to one BLS occupation code. In most cases, one O*NET code maps to one and only one BLS code, as in the following example:

| O*NET code | O*Net name | BLS code | BLS name |
| --- | --- | --- | --- |
| 11-3011.00 | Administrative Services Managers | 11-3011 | Administrative Services Managers |

In some cases, multiple O*NET occupations map to a single BLS occupation. In those cases, we take the average of an element’s values across the multiple O*NET codes and assign it to the single corresponding BLS code as in the following example:

| O*NET code | O*Net name | BLS code | BLS name |
| --- | --- | --- | --- |
| 11-3051.00  11-3051.01  11-3051.02  11-3051.03  11-3051.04 | Industrial Production Managers  Quality Control Systems Managers  Geothermal Production Managers  Biofuels Production Managers  Biomass Power Plant Managers | 11-3011 | Industrial Production Managers |

This leaves the two exceptions requiring further processing. O*NET version 24.2 still uses one occupation code that the was retired by the BLS after its 2016 data release: 25-3099 - Miscellaneous Teaching Occupations. In 2017 the BLS replaced this occupation code with two new codes, 25-3097 - Teachers and Instructors, All Other, Except Substitute Teachers, and 25-3098 - Substitute Teachers. O*Net 24.2 continues to use the older code. Therefore, first map O*Net values to the old BLS code 25-3099, and then apply those element averages to the two BLS occupations that replaced 25-3099, namely 25-3097 and 25-3098.

The full mapping is included in the accompanying file: bls_onet_crosswalk_2018.xlsx

**Supplemental References**

1. National Center for O*NET Development. O*NET OnLine. Available at: <https://www.onetonline.org/>; 2020

2. U.S. Bureau of Labor Statistics. Occupational Employment Survey. Available at: <http://www.bls.gov/oes/>; 2018
